# Small Economies in Cottage Hospital Management

**Published:** 1887-04-09

**Authors:** 

**Affiliations:** Oswestry Cottage Hospital


### Small Economies in Cottage Hospital Management.

By a Cottage Hospital Matron.

Please accept my grateful thanks for your note, which was an immense help to me. I felt reproached at seeing in The Hospital that you had noticed my apparent neglect, which indeed was not intentional, as I fully purposed writing. But our annual meeting was adjourned, and though it was for the ordinary committee meeting, as originally fixed, that I was anxious to have your opinion, as nothing definite could be settled till later, I waited for the result before writing to you again. Nor has any alteration been made, but six ladies are added to the committee, to act as visitors, I believe. The doctors' services are given, but they receive seven shillings for each patient, to pay for medicines, so that and the sum for drugs should be considered as one. I think there would not have been any question about my expenses if money had been forthcoming, but hospital work here labours under considerable disadvantage, as between the town of —— and village of —— there is a long-standing feud connected with it. Previous to my coming, this hospital had been managed by a lady in the village, and scarcely on hospital principles. It is wonderful what good was effected. Still, it not being satisfactory was the reason the change was made. When I came, there was not a medicine-glass in the place; other things about to match. Still, the committee have been very good in getting things, though there are two or three trying members. At the end of the second year, finding the general expenses in excess of income, and being reminded by outsiders what was done formerly under the old management, they wished for reduction to be made; and though I want to be as careful as possible, still, to have consented to the suggestions made, would simply have meant overworking those engaged, or leaving the work undone. So, having gained as much information as I could about other cottage hospitals, and with your valuable letter, I felt I was right in the matter, and had the committee insisted on the reductions, I should have resigned. But they do not seem to have entertained that idea, so I trust we shall go on comfortably now. I should like to take this opportunity of saying what an inestimable boon The Hospital is; and helpful as it cannot fail to be to all engaged in nursing work, it is, I think, more especially so to those who, like myself, are in a manner isolated from the hospital world, and living in a neighbourhood where some people seem to labour under the delusion that nurses must of necessity be related to Betsy Prigg and Mrs. Gamp. Thanking you once more for your letter, and with best wishes for the success of The Hospital.

---

By Miss Mattocks, Oswestry Cottage Hospital.

In the excellent article, "Workers *v.* Beggars," in The Hospital of February 5th, it is pointedly asked, How many of those who have administered the metropolitan and country hospitals have spent the money of the hospitals as they would have spent their own? If they had so spent it, would the hospitals have now been in such serious financial straits? I fear that from a very large number of hospitals the truthful answer to both questions would be, No! I remember that one of the first things which struck me in the surgical work of a large hospital was the quantities of things I thought then, and know now, to have been needlessly wasted. Bandages, as clean as when put on, were cut up every day; pieces of tissue, sometimes half a yard square, were put daily into the dust-pail, and all other things appeared to be used with a total disregard of cost. I am certainly under the mark in saying that the weekly waste of surgical materials in one ward alone would amount to one pound a week, or £52 a year. In ten wards the waste would amount to the income of many a small hospital, and in ten years it would have made a lump sum the managers of many an institution would now rejoice to see. Where nearly all the dressings are done by the surgeons and students, the former have alone the power of enforcing a careful use of things; but unfortunately the majority of men—young men especially—entertain a lordly contempt for small economies—the many littles that make a muckle—and lavishness in one thing encourages it in others. Why should I try to save pence for others to waste pounds? is a question likely to discourage those who would wish to be careful.

Unfortunately, the most irritating parsimony often accompanies reckless waste. I knew a matron of a provincial infirmary, otherwise an excellent and kind-hearted lady, who made a point of refusing everything she was not obliged to give. Though there were wards to be dusted and dishes to be washed every day, there were neither dusters nor dish-cloths furnished to the nurses, who therefore tore up rolls of lint for those necessary uses, with the result that what ought to have been a small item in the housekeeping accounts, made a large one in those of the dispensary department. In the same place I have seen carbolic oil poured on the fire to make it burn, just for want of a few sticks on each landing. Now for such evils I know but one remedy, the personal supervision of details by some one who knows what ought to be used, and is determined to permit no abuse. If every surgeon would avoid waste in dressing his own cases, if every ward sister or head nurse would be as careful in her ward as in her own house, if every matron or housekeeper would look sharply after the odds and ends so often wasted, our public institutions would be able to show a much greater amount of comfort for the same money as they now spend, or could show the same results as now at considerably less cost.

---

### WELCOME ALL!

Hospital, derived from *hospes*—guest, declares a welcome
to the suffering world;
With ever-growing skill, its flag of kindly care is never
furled;
Its lofty doors stand open wide, by night as well as day;
"Ye maimed, ye bruised, ye dying ones, ye poor, *this* is
your way!"—K. M.

